# p53-dependent expression of CXCR5 chemokine receptor in MCF-7 breast cancer cells

**DOI:** 10.1038/srep09330

**Published:** 2015-03-19

**Authors:** Nikita A. Mitkin, Christina D. Hook, Anton M. Schwartz, Subir Biswas, Dmitry V. Kochetkov, Alisa M. Muratova, Marina A. Afanasyeva, Julia E. Kravchenko, Arindam Bhattacharyya, Dmitry V. Kuprash

**Affiliations:** 1 Laboratory of Intracellular Signaling in Health and Disease, Engelhardt Institute of Molecular Biology, Russian Academy of Sciences, Vavilov str. 32, 119991 Moscow, Russia; 2 Department of Zoology, University of Calcutta, Ballygunge Circular Road 35, 700019 Calcutta, India; 3 Group of Regulation of Genome Transcription, Engelhardt Institute of Molecular Biology, Russian Academy of Sciences, Vavilov str. 32, 119991 Moscow, Russia; 4 Department of Immunology, Faculty of Biology, Lomonosov Moscow State University, Leninskye gory 1, 119234 Moscow, Russia

## Abstract

Elevated expression of chemokine receptors in tumors has been reported in many instances and is related to a number of survival advantages for tumor cells including abnormal activation of prosurvival intracellular pathways. In this work we demonstrated an inverse correlation between expression levels of p53 tumor suppressor and CXCR5 chemokine receptor in MCF-7 human breast cancer cell line. Lentiviral transduction of MCF-7 cells with p53 shRNA led to elevated CXCR5 at both mRNA and protein levels. Functional activity of CXCR5 in p53-knockdown MCF-7 cells was also increased as shown by activation of target gene expression and chemotaxis in response to B-lymphocyte chemoattractant CXCL13. Using deletion analysis and site-directed mutagenesis of the *cxcr5* gene promoter and enhancer elements, we demonstrated that p53 appears to act upon *cxcr5* promoter indirectly, by repressing the activity of NFκB transcription factors. Using chromatin immunoprecipitation and reporter gene analysis, we further demonstrated that p65/RelA was able to bind the *cxcr5* promoter in p53-dependent manner and to directly transactivate it when overexpressed. Through the described mechanism, elevated CXCR5 expression may contribute to abnormal cell survival and migration in breast tumors that lack functional p53.

CXCR5 (alternative name – Burkitt's lymphoma receptor 1 (BLR1)), is a G-protein coupled seven-transmembrane domain chemokine receptor^1^. Binding of CXCR5 to its ligand CXCL13 leads to activation of multiple intracellular signaling pathways which regulate cell proliferation, survival and migration^2^. Under normal conditions, CXCR5 is expressed by mature B cells and by follicular helper T cells and controls their migration into secondary lymphoid organs towards the gradient of CXCL13, produced by follicular stromal cells^3^,^4^. CXCR5 knockout in mice results in deficient lymphocyte traffic to the B cell areas of secondary lymphoid organs, with loss of proper homing of B lymphocytes to B cell zones^5^,^6^. The coordinated interaction between T helper cells and B cells is also lacking in CXCR5 deficient mice^7^.

Migration of malignant cells and leukocyte trafficking have many features in common^8^. Overexpression of chemokine receptors CXCR4, CCR3, CCR5, CCR7, CCR10 has been shown in breast cancer cell lines^9^,^10^,^11^. High levels of CXCR5 and CCR7 expression were also detected in primary breast tumors, and these levels correlated with metastatic and growth potential of the tumor^12^. Elevated expression of ligands for these receptors has been detected in organs and tissues which appear to be the metastasis destination^13^. In particular, an increase in CXCL13 mRNA has been observed in metastatic lymph nodes of breast cancer patients^14^, and significantly elevated serum CXCL13 in breast cancer patients displayed high correlation with tumor development and metastasis^15^. Recently, it was shown that CXCL13-CXCR5 co-expression in breast cancer patients highly correlates with lymph node metastases, suggesting CXCL13-CXCR5 axis as a potentially important therapeutic target in advanced metastatic breast cancer^16^. Other chemokine-receptor pairs have been linked to cancer as well, in particular the CXCL12-CXCR4 interaction^10^.

p53 is a tumor suppressor protein with prominent DNA-binding activity that can regulate expression of genes playing a significant role in cell cycle, apoptosis, senescence, or DNA-repair^17^. p53 activation takes place in response to a variety of oncogenic stress and DNA damage signals^18^. p53 mutations, most of which damage the p53 DNA-binding function, are found in more than half of all human cancers including breast tumors^19^,^20^. Many cancer cell lines also have defects and modifications in p53-dependent signaling pathways^21^,^22^. In breast cancer cells, p53 negatively regulates CXCR4 expression and decreases the level of tumor cell migration towards CXCL12 gradient^23^. The ability of p53 to repress expression of inflammatory chemokine receptors CCR2 and CCR5 was also shown in mice models^24^.

Transcription factors of NFκB family play critical role in inflammation, initiate the innate and adaptive immune responses and participate in activation of cell proliferation, growth, differentiation and survival^25^. Members of NFκB family can act as oncogenes and very often are constantly activated in tumor cells, contributing to malignant phenotype^26^.

In many cases, NFκB and p53 systems act as antagonists, respond to different types of stress and cannot function together in the same cell at the same time^27^. Known mechanisms of NFκB and p53 crosstalk and reciprocal regulation involve RAC-alpha serine/threonine-protein kinase (AKT-1 kinase), ADP-ribosylation factor 1 (ARF) and recruitment of p300/CBP coactivator^28^. There is some evidence that p53 can suppress NFκB directly which is consistent with p53 tumor suppressive function and with NFκB activation in p53-null tumors^23^. There is also data linking p53 loss to high levels of activated p65/RelA, a factor of NFκB family^29^.

Here we show that functional chemokine receptor CXCR5 is expressed in MCF-7 breast cancer cells. We determined that suppression of p53 leads to increased CXCR5 expression and activates cell migration in response to CXCL13. We also analyzed the *cxcr5* gene promoter activity and identified the promoter regions important for expression of the gene in breast cancer cells. Our data suggest an important role for NFκB both in basic *cxcr5* promoter activity and in its regulation by p53.

## Results

### p53 knockdown activates CXCR5 expression in MCF-7 cells

**Figure 1:**
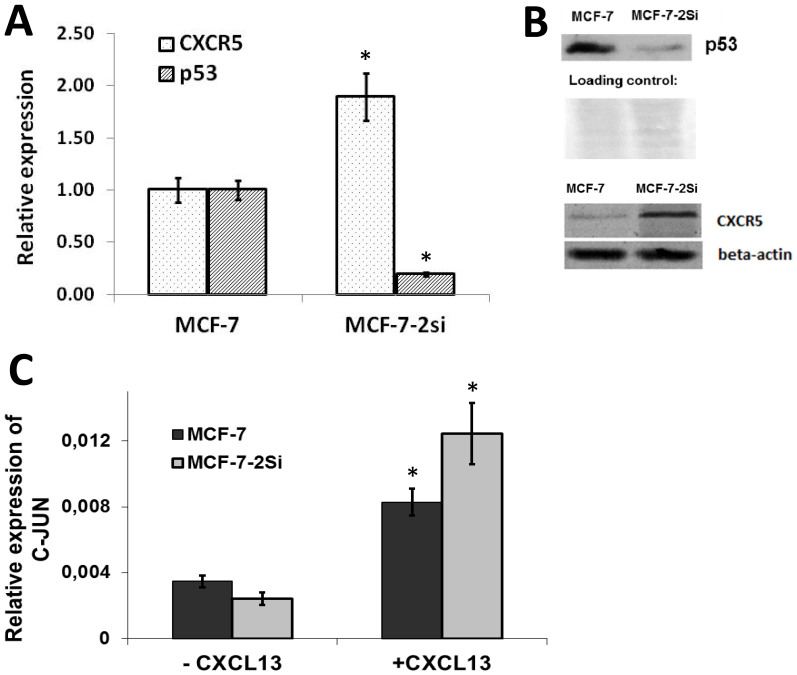
Inverse correlation between p53 and CXCR5 expression and function in MCF-7 cells. (A) p53 knock-down results in an increase in the relative abundance of CXCR5 mRNA. The result of 10 experiments is shown. (B) Changes in p53 and CXCR5 protein levels correlate with the levels of corresponding mRNAs. mRNA levels were measured by RT-PCR in real time. Representative data are shown, the experiment was repeated 2 times for p53 and 3 times for CXCR5. Complete western blots are shown in Supplementary Figure S1. (C) CXCR5 stimulates of c-Jun mRNA expression in MCF-7 cells in p53-dependent manner. Cells were exposed to recombinant CXCL13 for 6 hours prior to RNA isolation. The result of three experiments is shown. *P < 0,01 versus MCF-7.

MCF-7 human breast cancer cells express functional wild type p53^30^. Based on the existing indications of CXCL13-CXCR5 role in primary breast cancer cell migration^12^,^13^, we anticipated that CXCR5 may be expressed in cultured breast cancer cells. Indeed, using real-time RT-PCR and Western blotting analyses we detected CXCR5 mRNA and protein in breast cancer cell line MCF-7 (Figure 1A, B). We next asked whether p53 may downregulate CXCR5-mediated signaling pathways as part of its tumor suppressor function. We approached this question by p53 knockdown using lentiviral expression of a p53-specific short hairpin RNA in MCF-7 cells (Figure 1A, B). The transduced cell culture, termed MCF-7-2Si, demonstrated a significant increase in CXCR5 expression at both mRNA and protein levels as estimated by real time RT-PCR (Figure 1A) and Western blotting (Figure 1B) respectively. Thus, MCF-7 cells express CXCR5 in a p53-dependent manner. In contrast, suppression of non-functional mutant p53 in BT-20 cell line did not lead to any changes in CXCR5 expression (supplementary figure S2), indicating that functional DNA-binding activity of p53 is required for *cxcr5* gene regulation.

### Activation of CXCR5-dependent signaling pathways in MCF-7 cells inversely correlates with p53 status

CXCR5-CXCL13 interaction leads to transcriptional activation of a number of genes, including the gene encoding c-Jun transcription factor^31^. We utilized mRNA level of this gene as indicator of CXCR5 functional activity. The p53 knockdown had no effect on c-Jun expression (Figure 1C), while stimulation of MCF-7 and MCF-7-2Si cells with recombinant CXCL13 for 6 hours led to an increase in c-JUN which was significantly higher than the increase observed in parental MCF-7 cells (Figure 1C). Thus, elevated CXCR5 expression in MCF-7-2Si cells with p53 knockdown also translates into more robust CXCL13-dependent signaling.

### MCF-7-2Si cells demonstrate an increase in CXCL13-dependent chemotaxis

**Figure 2:**
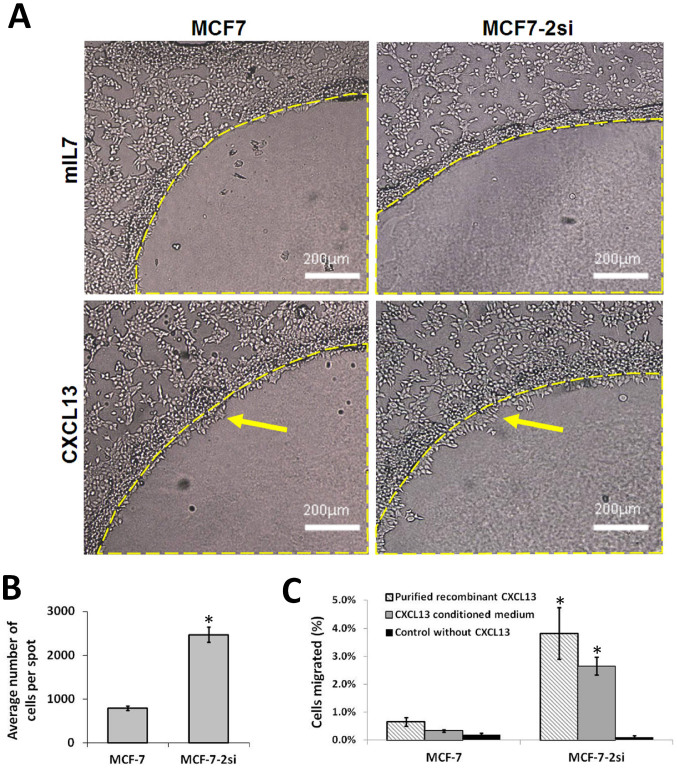
Increased CXCL13-dependent chemotaxis of MCF-7 breast cancer cells upon p53 knock-down. (A) MCF-7 cells did not migrate under the control spots containing IL7 (A, top left) and showed a low level of chemotactic activity towards recombinant CXCL13 (A, bottom left). MCF-7-2Si cells still showed no migration activity under control spots with IL7 (A, top right) but migrated under spots with CXCL13 more readily (A, bottom right). Yellow dashed lines show the borders of the spots, yellow arrows indicate the areas of cell migration. Similar results were obtained with purified recombinant CXCL13, however, the migration of MCF-7-2Si cells was even more aggressive (data not shown). (B) Average migration rates of MCF-7 and MCF-7-2Si cells under agarose spots were estimated by counting cells under 10 spots of similar radius. The experiment was repeated 6 times. (C) Chemotactic activity of MCF-7 and MCF-7-2Si cells in quantitative cell migration assay with ThinCert cell culture inserts. The data shown is the result of three replicate experiments. *P < 0,01.

Chemotactic potential of MCF-7 and MCF-7-2Si cells in response to CXCL13 was estimated using two alternative methods: an agarose spot assay for chemotactic invasion and a migration assay using ThinCert cell culture inserts. Both assays gave similar results that correlated with CXCR5 mRNA and protein levels (Figure 1) in the respective cells. MCF-7 cells showed a low level of migration activity towards recombinant CXCL13 while p53 knockdown in MCF-7-2si cells led to a significantly higher migration rate (Figure 2A–C). Both MCF-7 and MCF-7-2si cells demonstrated total lack of measurable migration activity towards recombinant interleukin 7 (IL7) (Figure 2A), indicating that the effect was CXCL13-specific. Thus, p53 modulates CXCL13-dependent migration activity of MCF-7 breast cancer cells.

### Identification of CXCR5 promoter and enhancer using bioinformatics

In order to determine potential mechanisms of p53-dependent *cxcr5* gene expression, we looked for regulatory elements in the *cxcr5* locus using available epigenetic information and UCSC Genome Browser. We took into account the number of regulatory histone modifications (H3K4me3, H3K4me1 and H3K27ac)^32^,^33^ and regions of DNase-I hypersensitivity^34^ in cells of non-hematopoietic origin, as well as high local concentration of predicted transcription factor binding sites (Supplementary Figure S3). According to our analysis, *cxcr5* gene promoter defined by current epigenetic data is similar to that reported earlier for HeLa cells^35^ and occupies the region from position −455 to +368 with respect to the transcription start site (TSS). There is also an apparent enhancer region in the first intron of *cxcr5* gene between +3.0 kb and +5.1 kb from the TSS.

### p53 downregulation in MCF-7 cells results in elevated *cxcr5* gene promoter activity

**Figure 3:**
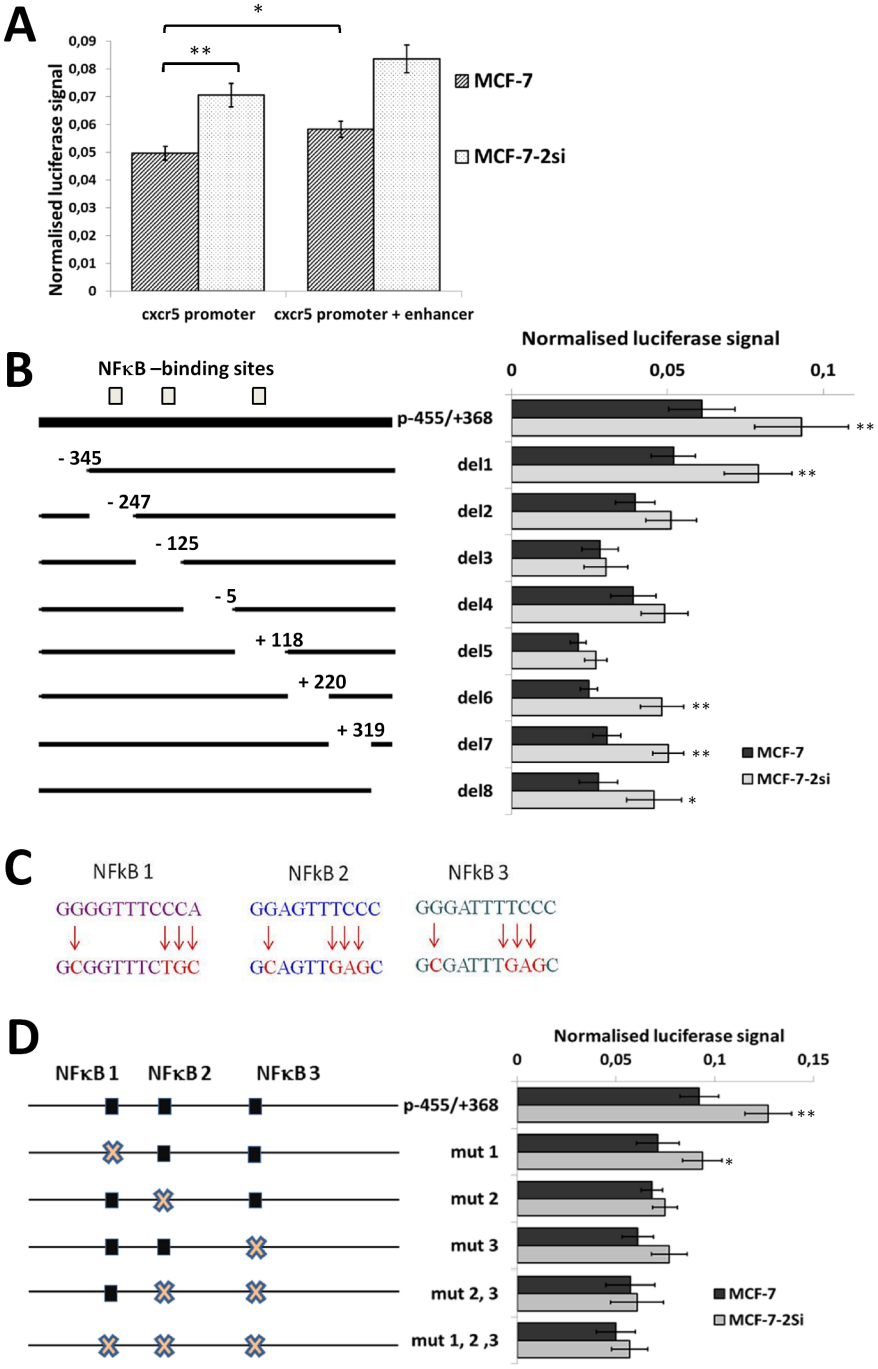
p53 downregulates *cxcr5* gene promoter activity via suppression of NFκB. (A) Suppression of p53 in MCF-7 cells led to 1.4-fold increase in *cxcr5* gene promoter activity while *cxcr5* gene enhancer demonstrated an additional modulating effect in both MCF-7 and MCF-7-2si cell lines. Data shown is the result of five replicate experiments. *P < 0,05; **P < 0,01. (B) Functional analysis of deletion scanning mutants of *cxcr5* promoter. Left, locations of the deletions on the promoter map. Positions of NFκB sites are shown. Right, normalized luciferase signals in MCF-7 and MCF-7-2si cells. *P < 0,05; **P < 0,01. (C) Sequences of predicted NFκB -binding sites in *cxcr5* promoter and nucleotide substitutions introduced by site-directed mutagenesis. (D) Functional analysis of the CXCR5 promoter mutants with different combinations mutations in NFκB sites. *P < 0,05; **P < 0,01.

Genomic fragment containing *cxcr5* promoter was subcloned upstream of luciferase gene in pGL3-Basic reporter vector. Another genomic fragment containing the potential *cxcr5* enhancer was subcloned downstream of the luciferase gene. In order to account for lower transfection efficiency of larger plasmids, the second construct included a control genomic fragment from *cxcr5* locus which was similar in length to the enhancer fragment but had no characteristic features of a regulatory sequence. Functional comparison of these two constructs in MCF-7 and MCF-7-2si cells (Figure 3A) revealed a marginal modulating effect by the enhancer (15%) towards *cxcr5* promoter activity. This effect, however, was stable in both cell lines, indicating that activity of the *cxcr5* intronic enhancer in breast cancer cells was independent of the p53 status. At the same time, the basic promoter activity in MCF-7-2si cells was 40% higher than in MCF-7 cells. Since there is no apparent p53 consensus binding site in *cxcr5* promoter, we hypothesized that p53 acts on *cxcr5* gene indirectly, utilizing other transcription factors.

### *cxcr5* promoter contains several distinct p53-responsive regions

We next designed 8 deletion variants (del1 −455/−345; del2 −345/−247; del3 −247/−125; del4 −125/−5; del5 −5/+118; del6 +118/+220; del7 +220/+319; del8 +319/+368) of *cxcr5* promoter (Figure 3B) to systematically identify the regions responsible for p53-dependent regulation of *cxcr5* promoter in breast cancer cells. All deletions, except for the most distal del1, led to a significant decrease in *cxcr5* promoter activity in both MCF-7 and MCF-7-2si cells. Importantly, deletion of regions 3 and 5 resulted in the complete loss of difference in the promoter activity between MCF-7 and MCF-7-2si cells. Therefore, these regions are likely to contain regulatory sequences responsible for p53-mediated effects on *cxcr5* gene activity. These regulatory elements appeared to act synergistically, since either deletion 3 or 5 alone completely abrogated the effect of p53 suppression on *cxcr5* promoter activity. Deletions 2 and 4 demonstrated partial effect.

### p53 modulates *cxcr5* promoter activity via suppression of NFκB activity

Transcription factors of NFκB family appear to be the main functional antagonists of p53, and p53 is able to suppress expression of some NFκB-dependent genes^27^. We hypothesized that the same mechanism could be responsible for *cxcr5* gene regulation. Using matrix-based nucleotide profiles of the transcription factors binding preference represented in JASPAR database^36^, we predicted three NFκB-binding sites in human *cxcr5* promoter. Sites at positions −274, −133 and +45 (Figure 3C) returned scores of 9.8, 10.6 and 12.8, respectively (typical for moderate affinity binding) and matched the NFκB sites described previously for the *cxcr5* promoter in B cells^35^. NFκB sites 2 and 3 lay within deletions 3 and 5, which correlated well with the results of deletion scanning analysis (Figure 3B). We then generated variants of *cxcr5* promoter with mutations of NFκB sites using nucleotide substitutions previously characterized by Wolf and colleagues^35^ alone and in combination and tested them in MCF-7 and MCF-7-2si cells (Figure 3D). The effect of NFκB1 mutation was similar to that of the region del2 containing NFκB1 site: the promoter activity was reduced while the activity in MCF-7-2si cells was still marginally stronger. Mutations of both NFκB2 and NFκB3 sites resulted in a more substantial reduction of promoter activity in MCF-7-2si cells, leading to a complete loss of sensitivity to p53 suppression (Figure 3D). Promoter variants with pairwise mutation of NFκB2 and NFκB3 sites, or all three NFκB sites together were even less active and demonstrated no significant difference between MCF-7 and MCF-7-2si cells (Figure 3D). Thus, p53 indeed acts upon human *cxcr5* gene via NFκB sites in *cxcr5* promoter.

### p65/RelA can directly transactivate *cxcr5* promoter

**Figure 4:**
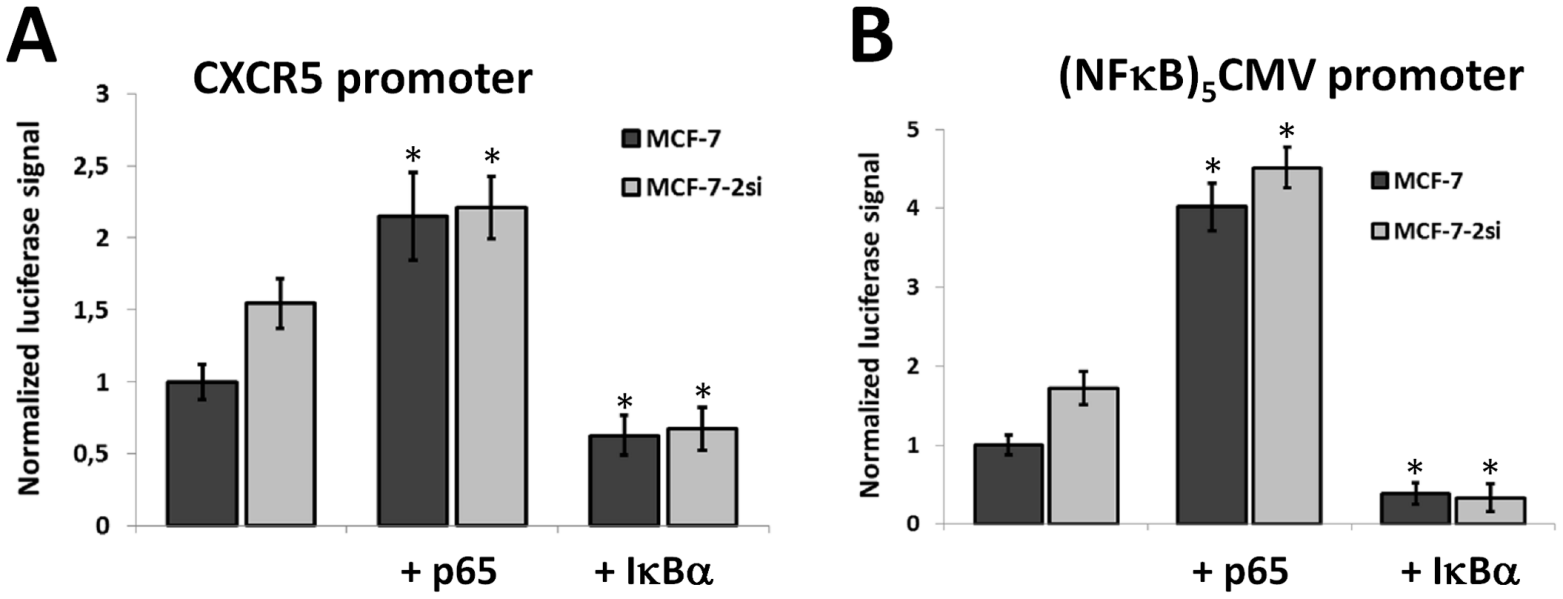
Changes in NFκB activity modulate *cxcr5* promoter activity. (A) Hyperactivation of NFκB by p65/RelA overexpression activates CXCR5 promoter in both MCF-7 and MCF-7-2si cells while NFκB inhibition by dominant-negative IκBα results in strong reduction of promoter activity in both cell lines. (B) NFκB reporter construct demonstrates the same pattern of responses to NFκB hyperactivation and inhibition as the CXCR5 promoter. The result of 3 experiments is shown. *P < 0,01.

To ascertain that the effect of p53 on CXCR5 expression is mediated by NFκB, we modulated the activity of NFκB pathway by transfecting the MCF-7 and MCF-7-2si cells either with p65/RelA or with dominant-negative IκBα. We then accessed the activity of the co-transfected luciferase reporter gene driven either by *cxcr5* promoter (Figure 4A) or by minimal CMV promoter and five copies of NFκB consensus site (Figure 4B). Both reporter constructs demonstrated elevated activity in MCF-7-2si cells as compared to MCF-7 cells, high activity in both cell lines overexpressing p65 and reduced activity in either cell line expressing dominant-negative IκBα (Figure 4). Of note, both p65-induced and IκBα-inhibited reporter activity of either of the two reporters was no longer p53-dependent (Figure 4). The model NFκB reporter responded to NFκB modulation more vigorously, presumably due to higher number and affinity of the NFκB sites. However, the pattern of activity was virtually the same for both reporter constructs (Figure 4), indicating that *cxcr5* is a *bona fide* NFκB-dependent gene.

### p65/RelA binds *cxcr5* promoter in p53-dependent manner

**Figure 5:**
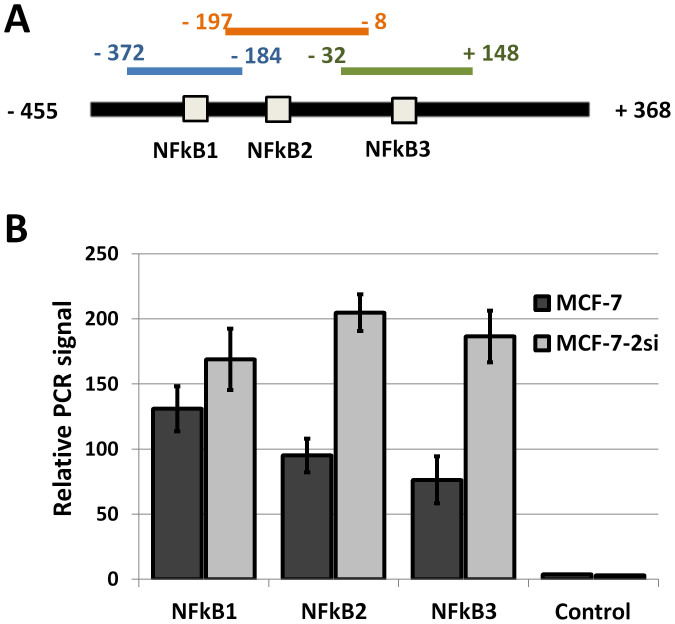
p53 suppression leads to elevated NFκB binding to *cxcr5* promoter *in vivo*. (A) Map of *cxcr5* promoter, predicted NFκB-binding sites and PCR products amplified in ChIP assay. (B) Suppression of p53 in MCF-7 cells leads to elevated p65/RelA crosslinking to the amplicons containing NFκB consensus sites 2 and 3.

To verify NFκB binding to *cxcr5* promoter *in vivo*, we used classical chromatin immunoprecipitation assay. We designed 3 amplicons from human *cxcr5* promoter, each comprising one of the three NFκB sites in the promoter (Figure 5A) and analyzed the amount of cross-linked DNA precipitated from MCF-7 and MCF-7-2si cells with anti-p65 antibodies (Figure 5B). Amplicon containing NFκB site 1 was similarly represented in the p65-crosslinked DNA precipitated from MCF-7 and MCF-7-2si cells (Figure 5B). However, amplicons containing NFκB sites 2 and 3 produced much stronger signals in MCF-7-2si cells, indicating elevated p65 binding upon p53 knockdown. These data correlate well with the results of *cxcr5* promoter studies and further confirm the role of NFκB in p53-dependent CXCR5 regulation.

## Discussion

p53 gene is frequently mutated in human cancers^19^. Loss of wild type p53 displays high correlation with invasive stages of tumor development^37^. Our data on p53-mediated CXCR5 repression illuminates one more possible mechanism of p53-dependent tumor suppression. Our data are in line with recent observation that co-expression of CXCL13 and CXCR5 in breast cancer patients closely correlates with tumor progression and lymph node metastasis^16^. Thus, CXCR5 expression may be closely connected to migration potential of breast cancer cells, similar to the well documented example of CXCR4^38^. Among other tumor suppressor functions, p53 was shown to suppress inflammatory microenvironment, with loss of p53 leading to activation of genes associated with chemotaxis and inflammation such as IL1, IL6, IL11, Ptgs2 and a number of chemokines^22^,^29^. Our observation that suppression of p53 in breast cancer cells promotes chemotaxis towards CXCL13 gradient, harmonizes with other p53 anticancer effects and fits the classical model of metastasis which involves immune cells that prepare tumor microenvironment for cancer cell migration^39^.

Since we did not find any p53-binding sites in *cxcr5* promoter, we concluded that mechanism of p53 action on *cxcr5* gene activity should be indirect, similar to that previously shown for the cxcr4 gene^23^. Therefore, we looked more closely at NFκB which is very often upregulated in cancer cells and antagonizes p53 in many ways. According to MCF-7 transcriptome profile from the CCLE database^40^, p65/RelA and p50/NFκB1 were identified as the most represented NFκB proteins in these cells (data not shown). The phenomenon of antagonism between components of the classical NFκB pathway and p53 has been studied in a number of experimental systems^27^, including direct demonstration that p53 suppression leads to increased p65 activity in cancer cells^29^,^41^. Indeed, *cxcr5* promoter contains several sequences similar to NFκB consensus that bind p65/RelA according to our ChIP results (Figure 5) and initiate transcription of a reporter gene in an NFκB-dependent manner (Figure 4). Importantly, point mutagenesis of NFκB sites completely abolishes the effect of p53 modulation on *cxcr5* promoter (Figure 3D). The effect is moderate but reproducible and statistically significant, and appears to operate exclusively through NFκB. We noticed that deletion mutagenesis leads to more intensive decrease in *cxcr5* promoter activity than the site-directed mutation of the predicted NFκB sites (Figures 3B and 3D). This may be due to the presence of binding sites for other transcription factors within the deleted promoter regions. Search of Jaspar database predicts a number of various binding sites within these regions of the promoter, including AP-1, Sp-1 and Ets-1 (data not shown).

It is known that p53 is able to suppress activity of pro-oncogenic transcription factors of AP-1 family, such as c-jun and ATF-1, by removal of p300 coactivator^42^. It can also repress activity of AP-1 and Ets-1 transcription factors by direct binding to them^43^,^44^. All these factors may be able to bind *cxcr5* promoter and modulate its activity. Clearly, any factors influencing the activity of *cxcr5* promoter may be mechanistically important for cancer progression as well as for other diseases involving immune pathologies. For example, SNP rs630923 associated with multiple sclerosis^45^ is located in the area of deletion 4 (Figure 3B), one of the regions required for the basal activity of the *cxcr5* promoter.

In summary, our data suggest that *cxcr5* is expressed in breast cancer cells as a part of the cell signaling system that regulates tumor cells survival, development and migration. This system is represented by transcription factors of NFκB as well as several other families which are all essential for optimal basal activity of the *cxcr5* promoter. This system is counterbalanced by the p53 protein which indirectly downregulates *cxcr5* expression in NFκB-dependent manner.

## Methods

### Cell lines

MCF-7 breast cancer cell line was kindly provided by late Dr. E. Zabarovsky from Karolinska Institute (Stockholm, Sweden). We recently authenticated our stock of MCF-7 cells by transcriptome profiling using Illumina HumanHT-12 v4 Expression Bead Chip and compared the data to available MCF-7 transcriptomes from GEO database (data not shown). MCF-7 cells were cultured in Dulbecco's modified Eagle's medium (DMEM, Life technologies, Carlsbad, USA) supplemented with 10% fetal bovine serum and 0.01% human insulin. HEK293 cells were cultured in Dulbecco's modified Eagle's medium supplemented with 10% fetal bovine serum.

### Ethical approval

Scientific Council of the Engelhardt Institute of Molecular Biology determined that experiments performed in this study did not require any ethical approval, because only commercially available cell lines were used.

### Suppression of p53 in MCF-7 cell line

HEK-293T cells were co-transfected with the lentiviral vector pLSLP-sh-p53-2 (kindly provided by prof. P. Chumakov, Engelhardt Institute of Molecular Biology RAS), envelope plasmid pVSV-G, and packaging vector pCMV-dR8.2. The supernatant containing the lentivirus particles was harvested at 48 h posttransfection, filtered through 0.22 μ membrane filter and used to infect 20% confluent MCF-7 cells in infection medium (DMEM) with 10% FBS and 8 mg/ml polybrene (Sigma, St. Louis, USA). Selection in the presence of puromycin (1 μg/ml, Sigma, St. Louis, USA) was carried out in the regular growth medium for 4 days. Initially, two different p53 shRNAs were tested. Both of them led to p53 suppression and to an increase in CXCR5 expression. Since shRNA-2 produced better p53 suppression (data not shown), it was used in all subsequent experiments. In a control experiment, MCF-7 cells were transduced with lentivirus containing GFP-expressing cassette which directed robust GFP expression but did not influence p53 expression or *cxcr5* promoter activity (supplemental figure S4).

### RNA extraction and real-time quantitative RT-PCR

Total RNA was isolated from cells using Trizol reagent (Invitrogen, Carlsbad, USA) following the manufacturer's protocol. RNA was reverse transcribed by M-MULV reverse transcriptase and oligo-dT primer from First strand cDNA synthesis kit (Fermentas, Vilnius, Lithuania). Quantitative RT-PCR was performed using Real-time PCR reaction mix with SYBR Green (Syntol, Moscow, Russia) and Applied Biosystems 7500 real-time PCR machine. The primer sequences used to amplify human β-actin, CXCR5, p53, NFATc3 and c-Jun cDNA are represented in Supplementary Table 1. The PCR program included preheating stage at 95°C for 10 min, followed by 40 cycles of amplification at 95°C for 15 s, 63°C for 20 s, 72°C for 20 s. The specificity of amplification products was controlled with the help of melting curve analysis. mRNA levels in all samples were normalized to β-actin.

### Recombinant chemokines and cytokines

CXCL13 and IL7 genes were amplified from genome DNA using primers containing XbaI and KpnI restriction sites (see Supplementary Table 1). CXCL13 and IL7 genes were cloned into a pcDNA3.1 vector using XbaI and KpnI restriction sites. HEK293 cells were transiently transfected with CXCL13 or IL7 vectors by calcium phosphate method using ProFection Mammalian Transfection System (Promega, Madison, USA) according to manufacturer's directions. Conditioned culture medium was collected 48 hours post transfection. Purified recombinant CXCL13 was purchased from Thermo Scientific (Waltham, MA, USA) and used at final concentration of 500 ng/ml.

### Detection of chemotactic activity using agarose spot assay for directed cell migration

The assay was performed essentially as described^46^. Briefly, CXCL13 or control IL7 conditioned culture medium was mixed with low-melting point agarose to final concentration of 0.5%. These agarose spots were placed on glass cover slips in a 6-well tissue culture plate and left at 4°C for 5 min to let agarose spots jellify. Subsequently, 2 ml of trypsinized cell suspension (2 million cells per ml) was added per well and the plate was incubated for 12 hours before analysis.

### Detection of chemotactic activity using quantitative cell migration assay

The assay was performed using ThinCert 8 μm pore cell culture inserts (Greiner Bio-One, Frickenhausen, Germany) was performed according to manufacturer's protocol. Both CXCL13 conditioned culture medium and purified recombinant CXCL13 were tested usign this assay, with similar results. The migrating cells were counted using Thiazolyl Blue Tetrazolium Bromide (MTT) (Sigma, St. Louis, USA) according to manufacturer's protocol. Migrated viable cells were quantified using MTT test, with MTT incubation for 24 hours followed by incubation with solubilizing solution for 3 hours.

### Analysis of transcriptional response to CXCL13

Cells were incubated in 50% conditioned medium containing CXCL13 or IL7 (control), 45% DMEM and 5% FCS for 6 hours, then total RNA was isolated and c-Jun mRNA levels were analyzed by RT-PCR in real time.

### Western blot analysis

Total cell lysates were prepared using 5× Laemmli buffer. Protein samples were resolved on 12% SDS-PAGE and transferred to Hybond-C Extra nitrocellulose membrane (Amersham Biosciences, Amersham, UK) and immunoblotted with anti-CXCR5 antibodies (MAB190, R&D Systems, Minneapolis, USA) at 1:3000 dilution, anti-p53 antibodies (2524 s, Cell Signaling Technology, Danvers, MA, USA) at 1:2000 dilution or anti-β-actin antibodies (1E5, Cell Signaling Technology, Danvers, MA, USA) at 1:3000 dilution. The bands were detected with ECL using SuperSignal West Dura Extended Duration Substrate (Thermo Scientific, Waltham, USA). β-actin expression and/or Ponceau membrane staining serve as loading control.

### Luciferase vector construction, site-directed mutagenesis and deletion scanning

The human *cxcr5* promoter (−455/+368) and enhancer element (+2991/+5107) were amplified by PCR using genomic DNA from MCF-7 cells as a template and specific primers (see Supplementary Table 1) containing cloning sites. CXCR5 promoter variants containing deletions and mutations in the predicted NFκB sites were generated by two-step PCR mutagenesis and verified by sequencing. All variants of CXCR5 promoter were digested with HindIII and NcoI, cloned into pGL3-basic luciferase reporter construct (Promega, Madison, USA), and sequenced. Predicted *cxcr5* gene enhancer element was cloned downstream of the luciferase gene using BamHI and SalI restriction sites.

NFκB response element consisting of 5 tandem NFκB consensus sites: GAG CTC GGG AAC TTC CGG GAA TTT CCG GGG AAG TCC GGG AAA TTC CGG GAC TTC CCC CCG GG, and minimal CMV promoter^47^ amplified from pEGFP-N3 plasmid (Clontech Laboratories, Madison, USA) with primers represented in Supplementary Table 1, were cloned into pGL3-basic luciferase reporter vector (Promega, Madison, USA) using restriction sites XhoI/HindIII and HindIII/NcoI respectively. Hyperactivation or inhibition of NFκB in MCF-7 cells was achieved using co-transfection of plasmid vectors expressing p65/RelA or dominant negative IκBα mutant^48^.

### MCF-7 cell transfection and luciferase reporter assay

Cells were transfected with 2 μg of purified plasmid DNA (combined amount per experiment for all test constructs) plus 100 ng of pRL-CMV Renilla luciferase control reporter vector (Promega, Madison, USA). DNA was incubated with 15 μg/ml of polyethylenimine (PEI) transfection agent^49^ for 20 minutes and then added to MCF-7 cells cultured in 6-well plate in 50% confluence. Luciferase activity was measured in Luminometer 20/20^n^ (TurnerBioSystems, Sunnwale, USA) using Dual-Luciferase Reporter Assay System (Promega, Madison, USA) following manufacturers protocol.

### Chromatin immunoprecipitation assay

We followed Cross-linking chromatin immunoprecipitation (X-ChIP) protocol by Abcam. Lysates with cross-linked with 0,75% formaldehyde protein-DNA complexes from 2 × 10^7^ MCF-7 cells were sonicated to obtain average DNA fragment size of 700 bp and incubated with antibodies to p65/RelA (D14E12, Cell Signaling Technology, Danvers, MA, USA) and precipitated with pre-blocked protein A sepharose beads. After elution of protein-DNA complexes and DNA purification, target DNA was quantified by real-time PCR. Three types of controls were used: background control without lysate; nonspecific precipitation control without antibodies; and an amplicon from a nonspecific locus containing no NFκB-binding sites (Supplementary Table 1). The primer sequences are represented in Supplementary Table 1.

### Statistical analysis

Statistical analyses were performed using Microsoft Excel and Statistica software. Statistical significance was determined using Mann–Whitney U test.

## Author Contributions

N.A.M. carried out most of the experiments, analyzed the data, participated in study design and drafted the manuscript. A.M.S. contributed to study design and participated in real time PCR analysis, chemotactic tests and bioinformatical analysis. C.D.H. initiated the study, carried out lentiviral suppression of p53 in MCF-7 cells, participated in real time PCR analysis and drafted parts of the manuscript. A.M.M. participated in promoter analysis and design of mutant promoters. D.V. Kochetkov and J.E.K. provided important help with lentiviral transduction. S.B. performed western blot analysis. M.A.A. provided important help with chemotaxis experiments and with data analysis. A.B. participated in study design and revised the manuscript. D.V. Kuprash supervised the study, analysed the data and revised the manuscript.

## Supplementary Material

Supplementary Information Supplementary Information
