# Supplementary material for: p53-dependent expression of CXCR5 chemokine receptor in MCF-7 breast cancer cells
Source: Sci Rep. 2015 Mar 19;5:9330. doi: 10.1038/srep09330 (PMC4365401; doi:10.1038/srep09330)
Supplement: Supplementary Information [file srep09330-s1.pdf]

Supplementary Material for:

p53-dependent expression of CXCR5 chemokine receptor in MCF-7 breast cancer cells

Nikita A Mitkin[1], Christina D Hook[1], Anton M Schwartz [1], Subir Biswas[2], Dmitry V Kochetkov[3], Alisa M Muratova[1,4], Marina A Afanasyeva[1], Julia E Kravchenko[3], Arindam Bhattacharyya[2], Dmitry V Kuprash [1,4*]

[1] Laboratory of Intracellular Signaling in Health and Disease, Engelhardt Institute of Molecular Biology Russian Academy of Sciences, Vavilov str. 32, 119991 Moscow, Russia

[2] Department of Zoology, University of Calcutta, Ballygunge Circular Road 35, 700019 Calcutta, India

[3] Group of Regulation of Genome Transcription, Engelhardt Institute of Molecular Biology Russian Academy of Sciences, Vavilov str. 32, 119991 Moscow, Russia

[4] Department of Immunology, Lomonosov Moscow State University, Leninskye gory 1, 119234 Moscow, Russia

* Corresponding author. Laboratory of Intracellular Signaling in Health and Disease, Engelhardt Institute of Molecular Biology Russian Academy of Sciences, Vavilov str. 32, 119991 Moscow, Russia. Phone: +7-499-135-9770; E-mail: kuprash@eimb.ru

Table of Contents: page

Supplementary Table 1 2

Supplementary Figure 1 4

Supplementary Figure 2 4

Supplementary figure 3 5

Supplementary figure 4 6

Table 1: sequences of oligonucleotides used in the work

Primers for Real-time PCR	
β-actin fw	5'-TGCGTGACATTAAGGAGAAG
β-actin rev	5'-GTCAGGCAGCTCGTAGCTCT
CXCR5 fw	5'-GCTAACGCTGGAAATGGA
CXCR5 rev	5'-GCAGGGCAGAGATGATTT
p53 fw	5'-ACTTTGCGTTCGGGCTGGGA
p53 rev	5'-GTCTGGCTGCCAATCCAGGGA
NFATc3 fw	5'- CCAAAGCCTGGCCACACCCC
NFATc3 rev	5'-TGCCCCTCGGCTACCTTCAGT
c-Jun fw	5'-TGGCAGAGTCCCGGAGCCAA
c-Jun rev	5'-CTCGCGCTCGCCCAAGTTCA

Primers for amplification of CXCL13 and IL7 genes from human genome	
CXCL13 fw	TTTGGTACCATGAAGTTCATCTCGACATCT
CXCL13 rev	AAATCTAGATCAGGGAATCTTTCTCTTAAACACT
IL7 fw	TTTGGTACCATGTTCCATGTTTCTTTTAGATATAT
IL7 rev	AAATCTAGATTATATACTGCCCTTCAAAATTTTATT

Primers for amplification of CXCR5 promoter, enhancer and control sequence from human genome	
Prom fw	GGTAAGCTTCTCGGCCTTCCAAAGTTGATT
Prom rev	TAACCATGGAGAATTCCAGACAGGGCCTC
Enh fw	TATGGATCCAGAGCCTCCTTCCAACAGAA
Enh rev	TATGTCGACTACAAGCTGGAACCATGGGT
Contr fw	ACTGGATCCCATCTGGTTAGAAAGTGTCA
Contr rev	TGAGTCGACCTATAAATGATGAACCCAGG

Primers for deletion scanning of CXCR5 promoter	
del1 fw	GGTAAGCTTGTTTTGCCTCTGTTGGGCAA
del2 fw	GGTGGATCCGACTGGAATGGTTGATCACC
del2 rev	GTTGGATCCCCCCTTCCCTTCTCAGCTCA
del3 fw	GGTGGATCCATCAACCTGCTGACTTTGCG
del3 rev	GGTGGATCCCTGCCAAGGACTGAAAAGTC

del4 fw	GGTGGATCCTTATTGCCGGGAAAGAAGCT
del4 rev	GGTGGATCCTGAGGGAAACTCCTGATCAA
del5 fw	TAAGGATCCCAGGTCGCGGCCCTACTGCC
del5 rev	GGTGGATCCATTGTCTCAACTGGGGCCTA
del6 fw	GGTGGATCCTCAACATAAGACAGTGACCA
del6 rev	GGTGGATCCGCCTCACAACTCATCACTT
del7 fw	GGTGGATCCTGAGTAGACACGGGTAGCTT
del7 rev	GGTGGATCCAGAGGCTCCCCGCCAGGTGC
del8 rev	TAACCATGGACCAGGTCCTCCAGGTTCTC

Primers for site-directed mutagenesis of NFkB sites	
mut1 fw	GACTTAGCGGTTTCTGCAGTCAAGGGACT
mut1 rev	TTGACTGCAGAAACCGCTAAGTCCTGGCA
mut2 fw	TGATCAGCAGTTGAGCTCATCAACCTGCT
mut2 rev	GAGCTCAACTGCTGATCAACAGAGAACAG
mut3 fw	CTGTGGGCGATTTGAGCTCTTTCTTCAAA
mut3 rev	AGAAAGAGCTCAAATCGCCCACAGCATCA

Primers for CHIP assay	
NFkB1 fw	ACCTATCTTTTCTGAACTTGAG
NFkB1 rev	GCCACCTCCAAACCTTCATT
NFkB2 fw	GGTTTGGAGGTGGCTTGGAG
NFkB2 rev	GTCTCAACTGGGGCCTATGA
NFkB3 fw	ACTAGTCATAGGCCCCAGTTG
NFkB3 rev	CGTCTCCTGAGGCAGTAGGG
Control fw	GCCACTGTCACACAGCTACT
Control rev	GTGTTTGACAGACTGGCTGAA

Primers for minimal CMV promoter amplification and oligonucleotides for NFkB response element assembling	
minCMV fw	TGGAAGCTTTAGGCGTGTACGGTGGGAG
minCMV rev	GAGCCATGGCGGATCTGACGGTTCACTAA
NFkB resp fw	GAGCTCGGGAACTTCCGGGAATTTCCGGGGAAGTCC
NFkB resp rev	CCCGGGGGGAAGTCCCGGAATTTCCCGGACTTCCCC

Figure S1: Full-size images of western blots of CXCR5 (A), β-actin (B) and of p53 (C)

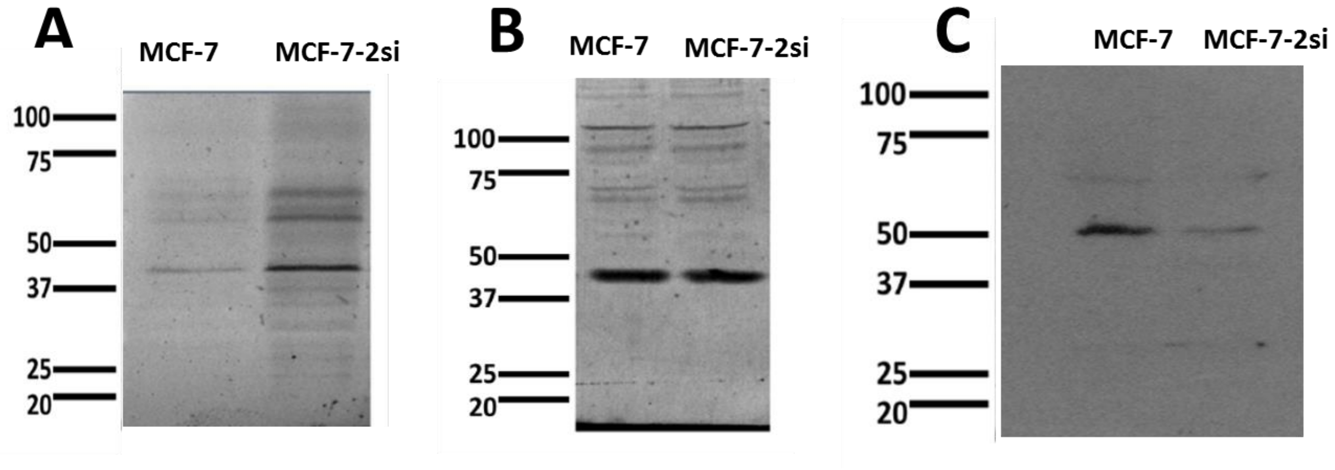

(A) and (B) are images of the same membrane stained with anti-CXCR5 and anti- β-actin, respectively.

Figure S2: Relative expressions of CXCR5 and p53 in breast cancer cell lines with wild type and mutant p53

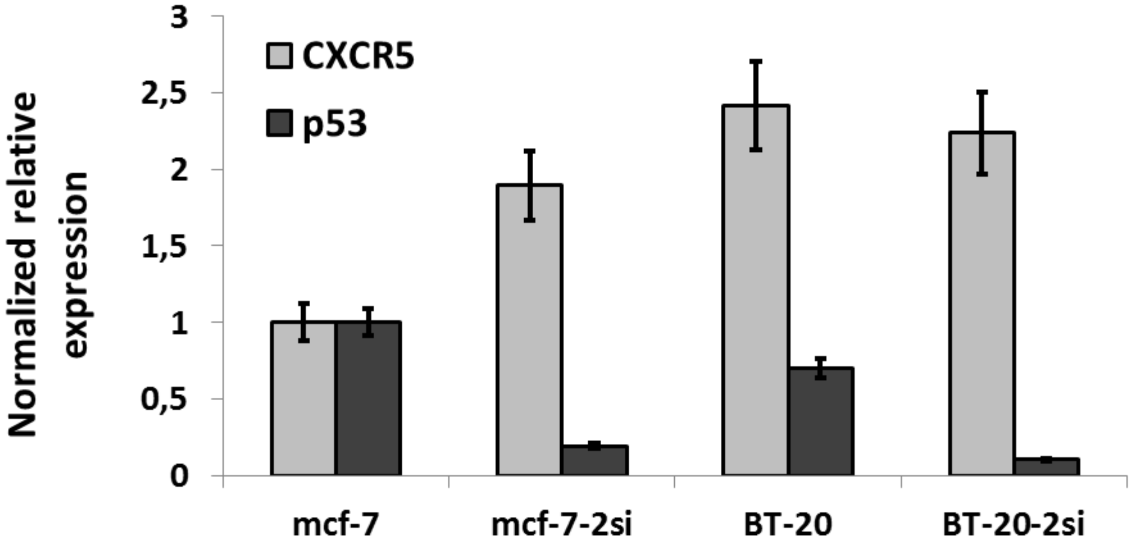

In BT-20 breast cancer cell line p53 is mutated and not functional as a DNA-binding protein. Basal expression of p53 in BT-20 cells is lower than in MCF-7. CXCR5 expression level in BT-20 is higher than in MCF-7-2si. High expression of CXCR5 in BT-20 correlates with p53 nonfunctional status. Suppression of mutant p53 in BT-20 cells (BT-20-2si) had no effect on CXCR5 expression.

Figure S3: Promoter and enhancer elements in cxcr5 gene.

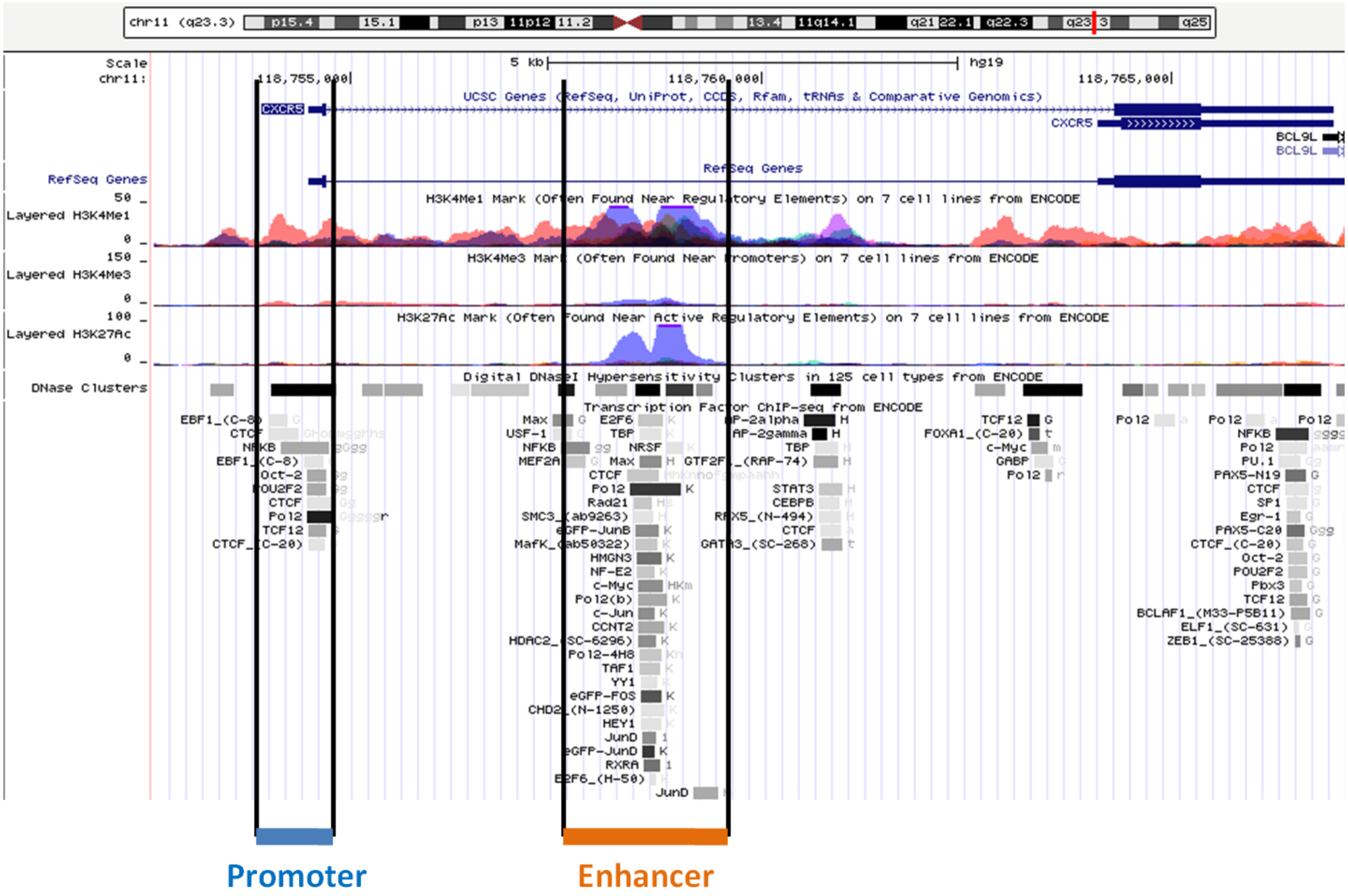

Scheme of cxcr5 gene (as displayed in UCSC Genome Browser) with maps of histone modifications and DNase I hypersensitivity clusters

Figure S4: Relative expressions of CXCR5 and p53 (A) and cxcr5 promoter activity (B) in original and lentivirus-transduced MCF-7 cells

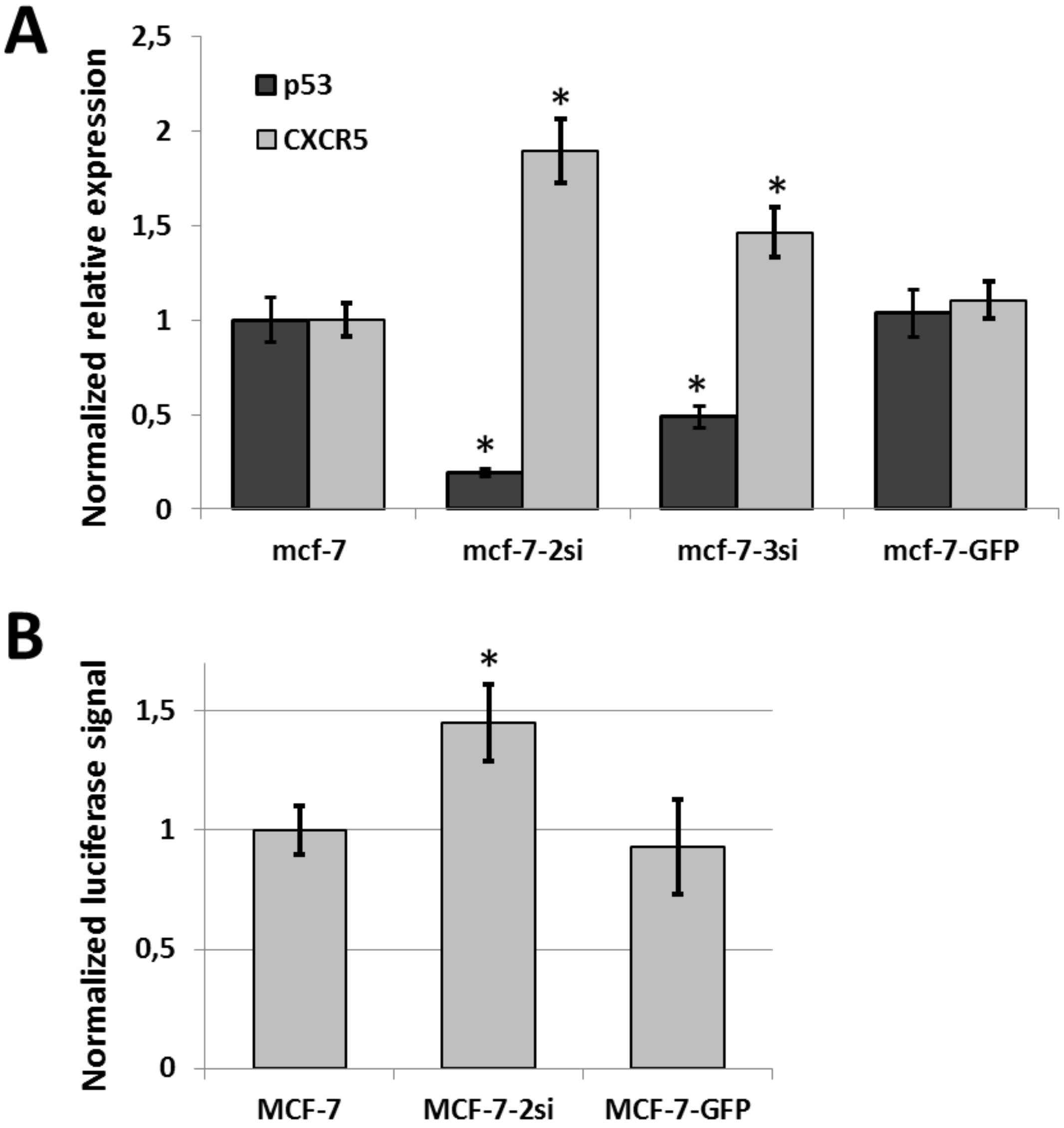

Cells transduced with two variants of p53 shRNA were named as MCF-7-2si and MCF-7-3si. The degree of shRNA-mediated p53 suppression correlates with CXCR5 expression level. Control MCF-7 cells were transduced with lentiviral vector containing GFP-expressing cassette which had no significant effect on p53 and CXCR5 expression levels (A) or on cxcr5 promoter activity (B).
